# A Rare Case Report of Dedifferentiated Endometrioid Carcinoma

**DOI:** 10.7759/cureus.56329

**Published:** 2024-03-17

**Authors:** Vallal Kani, Sumithra A, Jayaganesh P, Dhanya Menon

**Affiliations:** 1 Department of Pathology, Saveetha Medical College and Hospital, Saveetha Institute of Medical Sciences, Saveetha University, Chennai, IND

**Keywords:** abrupt transition, abnormal uterine bleeding, undifferentiated, endometrioid carcinoma, dedifferentiated

## Abstract

Dedifferentiated endometrioid carcinoma (DEC) is an exceptionally rare subtype of endometrial cancer characterized by a high-grade component juxtaposed with a low-grade endometrioid adenocarcinoma. This case report presents a unique instance of dedifferentiated endometrioid carcinoma in a 64-year-old female patient who presented with post-menopausal bleeding and abdominal pain. Diagnostic evaluation including imaging studies and histopathological examination revealed a mixed tumor comprising both high-grade and low-grade components. Management involved a multidisciplinary approach including surgical resection followed by adjuvant chemotherapy and radiation therapy. They are frequently mislabeled as endometrioid carcinomas of International Federation of Gynecology and Obstetrics (FIGO) Grade 2 or Grade 3. It is crucial to correctly differentiate these instances from traditional endometrioid carcinomas. This case underscores the importance of early recognition and comprehensive management strategies tailored to the unique characteristics of dedifferentiated endometrioid carcinoma. We report this case due to its rarity and complexity in diagnosis.

## Introduction

Dedifferentiation represents the existence of a high-grade tumour, which may develop from scratch, juxtaposed or recur after a previously well-differentiated tumour [1]. In a malignant neoplasm, dedifferentiation may serve as a histological marker of the tumour's advancement. This process also demonstrates the adaptability of some malignant neoplasms, as tumour cells shed their distinct characteristics and adopt less differentiated morphologies resembling the initial stages of embryonic growth or rejuvenating mechanisms [1,2]. Despite the lack of a clear incidence rate, dedifferentiated endometrioid carcinoma (DEC) was thought to be an uncommon subtype of endometrial cancer. On the other hand, it is known that the incidence percentage of undifferentiated endometrial carcinoma varies from 1 to 9% [2]. Furthermore, many retrospective analyses have shown that endometrial adenocarcinoma of low-grade type was associated with 37 to 87% of undifferentiated endometrial carcinoma. Dedifferentiated endometrioid carcinoma is frequently misinterpreted as International Federation of Gynecology and Obstetrics (FIGO) Grade 2 or Grade 3 endometrial cancer due to the simultaneous presence of a low-grade endometrial part along with an undifferentiated part [2]. Here we report a rare case of dedifferentiated endometrioid carcinoma in a 64-year-old female who came to our hospital.

## Case presentation

**Figure 1:**
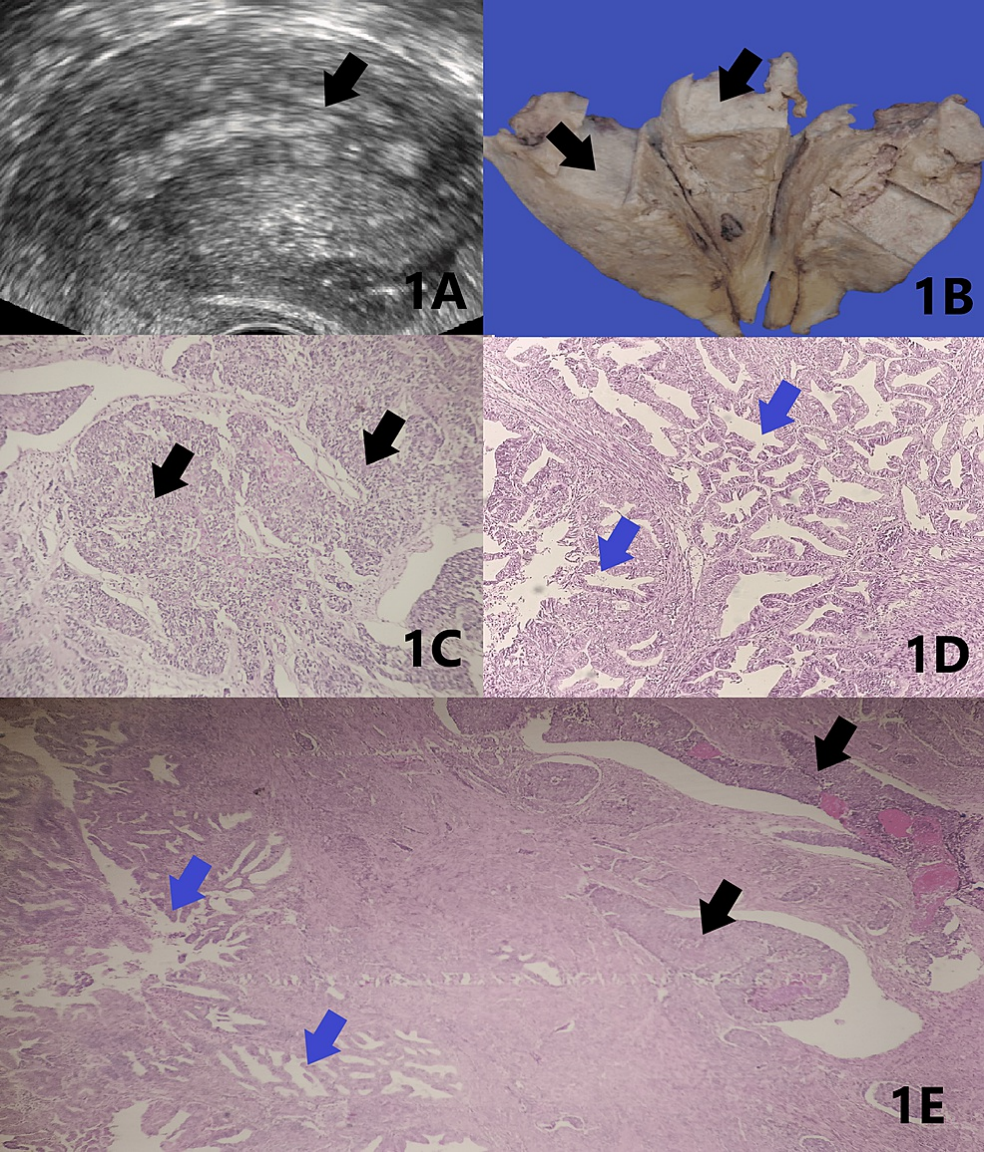
Ultrasonogram, gross and microscopy 1A: Ultrasonogram showing homogenous myometrium with poorly visualized endo-myometrial junction (arrow) 1B: Gross specimen showing cut surface with tumour (arrows) 1C: Undifferentiated component with solid sheets of tumour cells (black arrows) 1D: Well-differentiated component with glandular formation (blue arrows) 1E: Well-differentiated adenocarcinoma (blue arrows) juxtaposed with an abrupt transition of undifferentiated carcinoma (black arrows)

**Figure 2:**
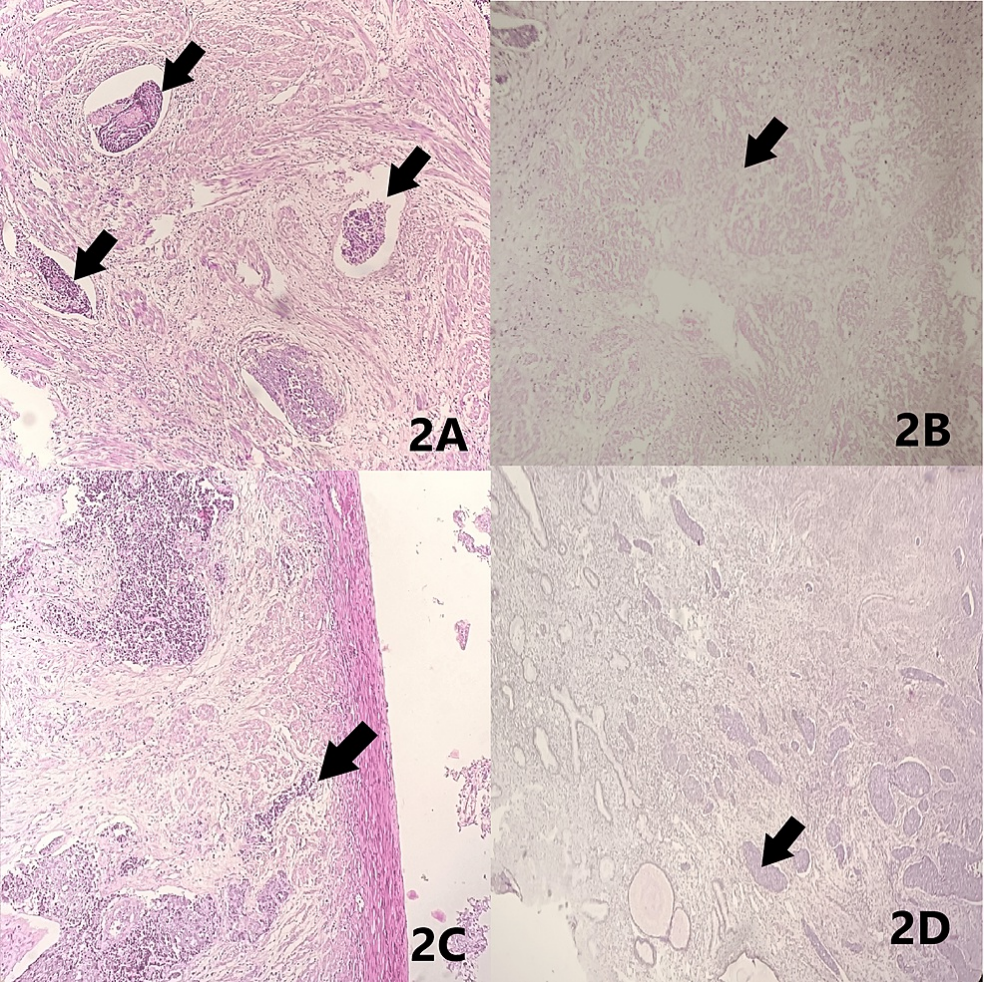
Microscopy 2A: Multiple lymphovascular invasions (arrows) 2B: Areas of necrosis (arrow) 2C: Tumor invading up to the uterine serosa (arrow) 2D: Tumor involving cervical stroma (arrow)

A 64-year-old female presented with post-menopausal bleeding, abdominal pain and per vaginal white discharge for two months. Imaging studies showed homogenous myometrium with poor visualization of the endo-myometrial junction (Figure 1A). Total abdominal hysterectomy with bilateral salpingo-oophorectomy was performed and the specimen was sent for histopathological examination. Macroscopically, we received a specimen of a uterus with a cervix measuring 9.5x5.5x3 cm. The endometrium showed an irregular grey-white tumour measuring 6x2.5 cm extending to the lower uterine segment. Grossly myometrial invasion appeared more than 50% (Figure 1B). The fallopian tubes measured 4.8 cm and 3.5 cm in length and the two ovaries measured 2x1x1 cm and 3x1.8x1 cm. Microscopy showed well-differentiated adenocarcinoma juxtaposed with an abrupt transition of undifferentiated carcinoma. The undifferentiated component showed solid sheets of monomorphic tumour cells with no differentiation (Figures 1C-1E). The tumor was myoinvasive with uterine serosal involvement invading the lower uterine segment and cervical stroma. Areas of lymphovascular invasion and necrosis were noted (Figures 2A-2D). No nodes were submitted. Based on the above findings, a diagnosis of dedifferentiated endometrioid carcinoma was reported. Pathologic stage was given according to pathological tumour-node-metastasis (pTNM), American Joint Committee on Cancer (AJCC) eighth edition as pT3a, pNx, and FIGO stage IIIA as the tumour invaded the serosa.

## Discussion

**Figure 3:**
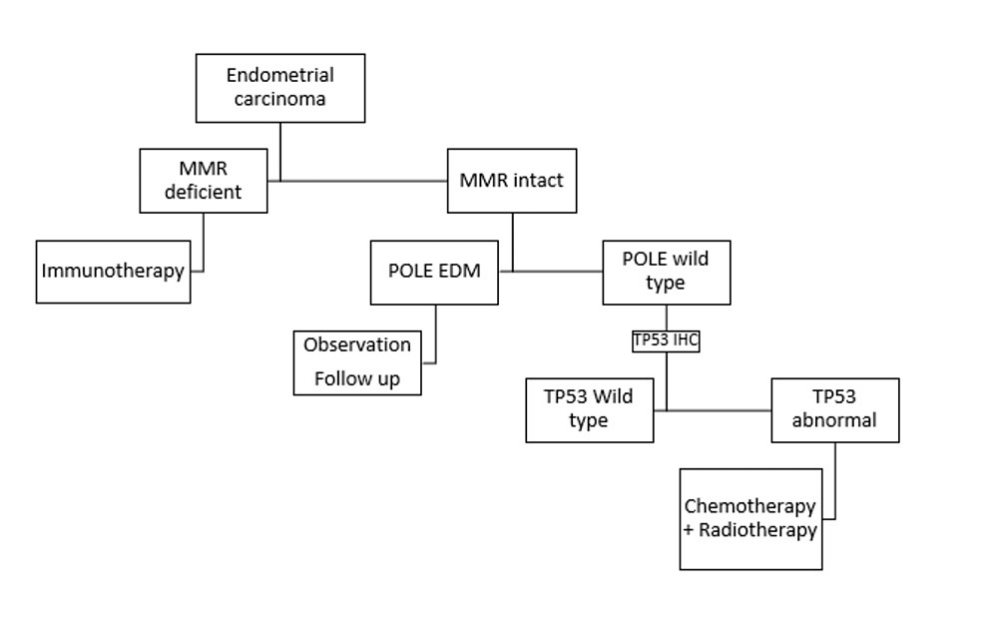
Schematic molecular classification of endometrial carcinoma MMR: mismatch repair, POLE: polymerase epsilon, IHC: immunohistochemistry, EDM: exonuclease domain mutations, TP53: tumour protein p53

DEC was first published by Tenti et al. in the year 1989 as a variant of well-differentiated endometrial carcinoma that had progressed to carcinoma of higher grade following chemotherapy [2]. After two decades, Silva et al. described dedifferentiated endometrioid carcinoma as a violent carcinoma marked by the concurrent existence of an undifferentiated and low-grade endometrial carcinoma in 2006 [3]. DEC is frequently misinterpreted as FIGO Grade 2 or Grade 3 endometrial cancer due to the simultaneous presence of a low-grade endometrial part along with an undifferentiated part. Even in cases where the undifferentiated component makes up only 20% of the total tumour, dedifferentiated endometrioid carcinoma has a worse prognosis, making the differentiation between these various entities crucial [2,3]. Dedifferentiation has been identified in several malignant epithelial neoplasms, including mucoepidermoid carcinoma, myoepithelial carcinoma, adenoid cystic carcinoma, and salivary gland carcinoma [2,4]. It is frequently linked to enhanced invasion of cancer cells and resistance to drugs. Furthermore, dedifferentiated carcinomas were also noted in the pancreas, urinary tract, and gastrointestinal tract [2,5]. As far as uterine endometrial cancers are concerned, endometrioid carcinoma is the most often detected histological form; clear cell, serous, undifferentiated, and especially dedifferentiated endometrioid carcinomas are very rare. Histologically, there is an abrupt transition between the two malignant components with a sharp distinct border. Moreover, the tumours of bone and soft tissues like chondrosarcoma, osteosarcoma, and liposarcoma have been observed to exhibit the same histological pattern. The clinical characteristics of the disease, including incidence, prognosis, and treatment, are unclear since the pathological characterization of dedifferentiated endometrioid carcinoma was only recently established [6]. It occurs most commonly in the sixth to seventh decades, but cases at age less than that are also reported [2]. Clinically, it is characterized by bleeding per vagina. In most instances, the diagnosis is made when metastasis already occurred. The metastases are encountered in the brain, bone, adrenals, etc [2,7]. In some cases, dedifferentiated endometrioid carcinoma is found to be associated with Lynch syndrome, which is an autosomal dominant disorder linked to germline mutations in the mismatch repair (MMR) genes [2]. Figure 3 represents schematic molecular classification of endometrial carcinoma using immunohistochemical and Sanger sequencing techniques to ascertain treatment plans and prognosis [2].

It is challenging to diagnose dedifferentiated endometrioid carcinoma preoperatively with solely endometrial curettage. The undifferentiated components of the tumour are visible deeper in the myometrium than the differentiated components, which is why a sufficiently big specimen sample is required to confirm the conclusive diagnosis of dedifferentiated endometrioid carcinoma [8]. Distinguishing the undifferentiated part from the endometrial carcinoma of higher grade is crucial for the diagnosis of dedifferentiated endometrioid carcinoma. Immunohistochemical tests are useful in performing differential diagnosis, and the point is whether or not there are areas of glandular formation. The keratins, epithelial membrane antigen (EMA), estrogen receptor (ER), and progesterone receptor (PR) are significantly positive in differentiated tumour areas, but the expression of these markers is nearly absent in undifferentiated tumour areas, or there is just focal staining for EMA and keratins [9]. When making a differential diagnosis for the undifferentiated component of dedifferentiated endometrioid carcinoma, the conditions to be taken into consideration are uterine carcinosarcoma, endometrial carcinoma of higher grade, neuroendocrine carcinoma, and unclassified sarcoma [10].

## Conclusions

The presented case report highlights the unique clinical and pathological features of dedifferentiated endometrioid carcinoma, emphasizing its rarity and challenging diagnostic considerations. Through comprehensive examination and multidisciplinary collaboration, a precise diagnosis was achieved, guiding tailored treatment strategies and underscoring the importance of individualized patient care in managing such uncommon malignancies. Despite the literature that is now accessible, some cases of aggressive behaviour of the tumour or advanced disease may likely prevent the diagnosis of uterine endometrial cancer with dedifferentiated endometrioid carcinoma, indicating that the incidence of dedifferentiated endometrioid carcinoma may be larger than that of prior research. As we work to enhance dedifferentiated endometrioid carcinoma diagnosis, it is equally important to evaluate prognosis and suitable treatment for individuals who already have a confirmed diagnosis of dedifferentiated endometrioid carcinoma.
